# Simultaneous multi-slice steady-state free precession myocardial perfusion with iterative reconstruction and integrated motion compensation

**DOI:** 10.1016/j.ejrad.2022.110286

**Published:** 2022-06

**Authors:** Sarah McElroy, Karl P. Kunze, Muhummad Sohaib Nazir, Peter Speier, Daniel Stäb, Adriana D.M. Villa, Momina Yazdani, Vittoria Vergani, Sébastien Roujol, Radhouene Neji, Amedeo Chiribiri

**Affiliations:** a School of Biomedical Engineering and Imaging Sciences, Faculty of Life Sciences and Medicine, King’s College London, London, United Kingdom; b MR Research Collaborations, Siemens Healthcare Limited, Frimley, United Kingdom; c Cardiovascular Predevelopment, Siemens Healthcare GmbH, Erlangen, Germany; d MR Research Collaborations, Siemens Healthcare Limited, Melbourne, Australia

**Keywords:** Myocardial perfusion, Simultaneous multi-slice, Motion compensation, *BH*, Breath-hold, *bSSFP*, Balanced steady-state free precession, CAD, Coronary artery disease, *CMR*, Cardiac magnetic resonance, *GC-LOLA*, Gradient-controlled local Larmor adjustment, *GRAPPA*, Generalized autocalibrating partial parallel acquisition, *ICC*, Intraclass correlation coefficient, *MC*, Motion compensation, *NMC*, No motion compensation, *SENSE*, Sensitivity encoding, *SMS*, Simultaneous multi-slice, *SNR*, Signal-to-noise ratio, *TE*, Echo time, *TR*, Repetition time

## Abstract

**Purpose:**

Simultaneous multi-slice (SMS) balanced steady-state free precession (bSSFP) acquisition and iterative reconstruction can provide high spatial resolution and coverage for cardiac magnetic resonance (CMR) perfusion. However, respiratory motion remains a challenge for iterative reconstruction techniques employing temporal regularisation. The aim of this study is to evaluate an iterative reconstruction with integrated motion compensation for SMS-bSSFP first-pass myocardial stress perfusion in the presence of respiratory motion.

**Methods:**

Thirty-one patients with suspected coronary artery disease were prospectively recruited and imaged at 1.5 T. A SMS-bSSFP prototype myocardial perfusion sequence was acquired at stress in all patients. All datasets were reconstructed using an iterative reconstruction with temporal regularisation, once with and once without motion compensation (MC and NMC, respectively). Three readers scored each dataset in terms of: image quality (1:poor; 4:excellent), motion/blurring (1:severe motion/blurring; 3:no motion/blurring), and diagnostic confidence (1:poor confidence; 3:high confidence). Quantitative assessment of sharpness was performed. The number of uncorrupted first-pass dynamics was measured on the NMC datasets to classify patients into ‘suboptimal breath-hold (BH)’ and ‘good BH’ groups.

**Results:**

Compared across all cases, MC performed better than NMC in terms of image quality (3.5 ± 0.5 vs. 3.0 ± 0.8, *P =* 0.002), motion/blurring (2.9 ± 0.1 vs. 2.2 ± 0.8, *P <* 0.001), diagnostic confidence (2.9 ± 0.1 vs. 2.3 ± 0.7, *P <* 0.001) and sharpness index (0.34 ± 0.05 vs. 0.31 ± 0.06, P < 0.001). Fourteen patients with a suboptimal BH were identified. For the suboptimal BH group, MC performed better than NMC in terms of image quality (3.8 ± 0.4 vs. 2.6 ± 0.8, *P <* 0.001), motion/blurring (3.0 ± 0.1 vs. 1.6 ± 0.7, *P <* 0.001), diagnostic confidence (3.0 ± 0.1 vs. 1.9 ± 0.7, *P <* 0.001) and sharpness index (0.34 ± 0.05 vs. 0.30 ± 0.06, P = 0.004). For the good BH group, sharpness index was higher for MC than NMC (0.34 ± 0.06 vs 0.31 ± 0.07, P = 0.03), while there were no significant differences observed for the other three metrics assessed (*P >* 0.11). There were no significant differences between suboptimal BH MC and good BH MC for any of the reported metrics (*P >* 0.06).

**Conclusions:**

Integrated motion compensation significantly reduces motion/blurring and improves image quality, diagnostic confidence and sharpness index of SMS-bSSFP perfusion with iterative reconstruction in the presence of motion.

## Highlights

• Motion compensation integrated into temporal regularization improves quality of SMS myocardial perfusion imaging.
• The integration of motion compensation does not degrade motion-free images.
• The presented framework will aid translation of high spatial coverage SMS-bSSFP perfusion imaging into clinical practice.

## 1 Introduction

Cardiac magnetic resonance (CMR) perfusion imaging is routinely used for ischemia assessment in patients with suspected coronary artery disease (CAD) and is currently recommended by international guidelines [1], [2].

CMR perfusion is typically performed using an ECG-triggered 2D multi-slice acquisition during the first pass of a contrast agent. Spatial coverage is typically limited to 3–4 slices using conventional CMR perfusion sequences, which precludes assessment of total ischemic burden [3]. Suboptimal planning may also result in missing parts of the myocardium in the acquired images, limiting the diagnostic value of the scan.

To improve the spatial coverage of cardiac MR perfusion scans, while maintaining high in-plane spatial resolution (<2 × 2 mm^2^) and sufficient temporal resolution (1 R-R interval), it is necessary to accelerate the image acquisition. 3D techniques have been proposed to improve spatial coverage but are often associated with reduced in-plane spatial resolution. Alternatively, simultaneous multi-slice (SMS) imaging [4], [5], [6] is an acceleration technique which enables greater spatial coverage by acquiring multiple slices simultaneously. SMS can be combined with CAIPIRINHA encoding, which introduces a slice-specific shift in the FOV, for improved slice separation using parallel imaging techniques [7]. This has been applied to first-pass myocardial perfusion with a balanced steady-state free precession (bSSFP) readout [8], [9], [10], [11] and can be combined with iterative reconstruction for improved image quality and improved perceived SNR compared with a conventional 3-slice bSSFP acquisition [12].

However, the temporal sparsity of the dynamic series, which is exploited in iterative reconstruction frameworks employing temporal regularisation, is reduced in the presence of motion. This sensitivity to respiratory motion is typically mitigated by asking patients to hold their breath during the first-pass of the contrast agent, which is not always a robust solution as it relies on correct timing of the breath-hold (BH) after contrast administration, and patients with cardiac conditions may struggle to maintain a BH for the duration of the first-pass. In the context of a reconstruction with temporal regularisation and without motion compensation, a suboptimal BH during the first-pass can result in blurring, artefacts and misalignment of dynamically acquired frames [13], [14], [15], [16], [17]. Furthermore, quantification of myocardial blood flow from CMR perfusion images requires motion correction across the perfusion series [18], [19]. Therefore, motion corruption remains a significant limitation preventing clinical adoption of such accelerated MR imaging techniques.

A recent study described the integration of non-rigid motion models into the iterative reconstruction with a temporal regularisation framework to reduce sensitivity to respiratory motion [15]. The goal of this study was to evaluate the applicability of such a motion-compensated iterative reconstruction framework for stress SMS-bSSFP first-pass myocardial perfusion in the presence of respiratory motion.

## 2 Materials and methods

### 2.1 Study population

Thirty-one patients (20 male, 11 female, mean age 58 ± 14 years) were prospectively recruited for the study based on a clinical suspicion of CAD. This included an assessment of chest pain or chest pain equivalent (for example shortness of breath) and the associated cardiovascular risk factors, which included hypertension, diabetes, smoking history and hypercholesterolaemia. Based on this clinical information, an assessment of possible CAD was undertaken with subsequent stress imaging as described in this study. The study was approved by the National Research Ethics Service (15/NW/0778) and written informed consent was obtained from all patients.

### 2.2 Data acquisition

All patients were scanned on a 1.5 T MR scanner (MAGNETOM Aera, Siemens Healthcare, Erlangen, Germany) using an 18-element body coil array and 32-element spine array. A SMS-bSSFP prototype sequence with gradient-controlled local Larmor adjustment (GC-LOLA) [9] was employed for perfusion imaging and planned on the systolic phase of 2-, 3- and 4-chamber cine acquisitions to prescribe 2 basal, 2 mid and 2 apical slices. Adenosine was administered for a minimum of 4 min prior to a single bolus of 0.075 mmol/kg of gadobutrol (Gadovist, Bayer, Berlin, Germany), which was administered at a rate of 4 ml/s, followed by a 20 ml flush of saline. Patients were instructed to hold their breath during the first-pass of the contrast agent, following current clinical guidance [20]. The following parameters were used for the acquisition: FOV: 360 × 360 mm^2^, TR/TE/FA: 2.9 ms/1.24 ms/50°, saturation time: 94 ms, pixel size: 1.9 × 1.9 mm^2^, slice thickness: 10 mm, multiband factor: 2, total acceleration factor: 7, readout duration per slice/slice group: 156 ms, bandwidth: 1302 Hz/ Px. The total number of dynamic frames acquired was 80 and the TGRAPPA scheme [21] was used for time-interleaved linear undersampling. Note that prior to entering the MRI scanner, patients were instructed to practice taking an expiratory BH for approximately 15 s, or as long as possible for each patient. This was practiced outside the scanner bore with a physician, in order to familiarise the patient with the expected BH during the MRI scan.

### 2.3 Image reconstruction

Image reconstruction was performed offline using a precompiled prototype C++ implementation of an iterative reconstruction framework with integrated motion compensation for SMS-bSSFP perfusion data. It is comprised of a non-rigid motion estimation step in combination with an iterative algorithm using temporal regularisation and motion compensation, similar to the technique reported in [15], where the non-rigid deformation fields between the dynamic frames are incorporated into the temporal regularisation term. In contrast to the approach described in [15], the basis for motion estimation was a separate, preliminary reconstruction step in which the data was resampled to a slightly lower resolution and reconstructed using a Conjugate-Gradient SENSE algorithm without any temporal regularisation, in order to preserve the fidelity of the depicted motion states across dynamics. In order to limit the impact of high-intensity signals such as chest fat and noise, histogram equalisation was performed on these preliminary images before performing a consecutive non-rigid registration [22]. The motion fields generated by the registration step were then incorporated into the final reconstruction as part of the temporal regularisation as described in [15]. Coil sensitivity maps for iterative reconstruction were calculated on the basis of a temporal average of measured raw data from all dynamics [15]. The employed SMS framework reconstructs the two simultaneously acquired slices for each SMS acquisition in a single image with a two-fold larger phase FOV [23]. Only motion estimation and the application of motion fields are performed separately for each of the two slices in the SMS image during reconstruction. In order to assess the effect of the integrated motion compensation on the iterative reconstruction, all datasets were reconstructed twice, i.e. with and without integrated motion compensation, leading to a motion-compensated (MC) and a non-motion-compensated (NMC) dataset for each patient case respectively.

### 2.4 Image analysis

The first-pass section of each dataset was independently assessed by three readers (AC, MSN and ADMV with 15, 5 and 7 years of CMR experience, respectively). The readers were blinded to the clinical details and as to whether the reconstruction was motion-compensated or not. Metrics assessed were image quality (1 = severe artifacts/non diagnostic, 2 = major artifacts but of diagnostic quality, 3 = minor artifacts and of diagnostic quality, 4 = excellent/no artifacts), motion/blurring (1 = significant blurring/motion, 2 = minor blurring/motion, 3 = no blurring/motion) and diagnostic confidence (1 = low diagnostic confidence, 2 = moderate diagnostic confidence, 3 = high diagnostic confidence). Additionally, a quantitative assessment of sharpness index was performed across the blood-myocardium interface at peak myocardial enhancement, as previously described [24], [25]. The analysis was performed on the third slice of each dataset (corresponding to a mid-ventricular slice), to limit any partial volume effects more commonly observed in basal and apical slices. Multiple closely spaced points were drawn at either side of the border between the LV blood pool and the septal wall. Each point in the septal wall was matched to the closest point in the LV blood pool and a signal profile was generated. The sharpness index was calculated for each signal profile as the reciprocal of the distance over which the signal intensity increased from 20% to 80% of the signal intensity range. The mean value across all profiles was then calculated as an average estimate of the sharpness index.

Comparisons between NMC and MC reconstructions were performed across all datasets, as well as within and between two subgroups of patients exhibiting either good or suboptimal BH. For classification of subgroups, the number of uncorrupted first-pass frames (i.e. number of BH frames after contrast appearing in basal slice of left ventricular blood pool) was counted on all datasets by reader 2 (MSN), and datasets with a suboptimal BH were identified as those with <20 uncorrupted first-pass frames on the respective NMC series.

### 2.5 Statistical analysis

For the global comparison as well as within each subgroup (datasets with a good BH and datasets with a suboptimal BH), the Wilcoxon signed-ranks test was used to compare the image quality, motion/blurring and diagnostic confidence scores between NMC and MC datasets, while the paired *t*-test was used to compare sharpness index. Between patient subgroups (good BH vs. suboptimal BH), the Mann-Whitney *U* test was used to compare qualitative metrics and the unpaired *t*-test was used to compare measurements of sharpness index. All tests were two-sided and values of *P* < 0.05 were considered significant. Inter-reader agreement for the three qualitative metrics was assessed using the two‐way mixed average measures intraclass correlation coefficient (ICC).

## 3 Results

**Fig. 1:**
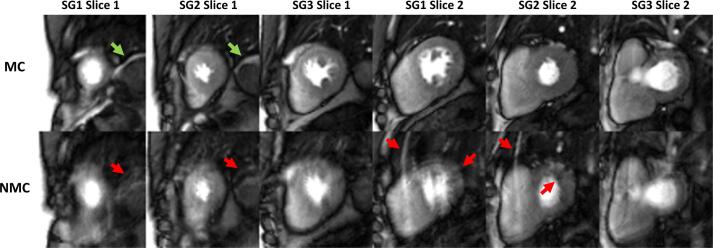
SMS-bSSFP perfusion images at peak myocardial enhancement acquired in a patient with a suboptimal BH (3 uncorrupted first-pass frames) and no apparent perfusion deficit. MC scored higher than NMC in terms of image quality (4.0 vs. 1.0), motion/blurring (3.0 vs 1.0) and diagnostic confidence (3.0 vs 1.0). For NMC, the red arrows highlight chest wall ghosting due to motion, as well as leakage of moving diaphragmatic fat between slices. The signal from diaphragmatic fat is restored in the MC images (green arrows), and ghosting from the chest wall is absent. See Supplementary data 1 for a video of the complete perfusion series.

**Fig. 2:**
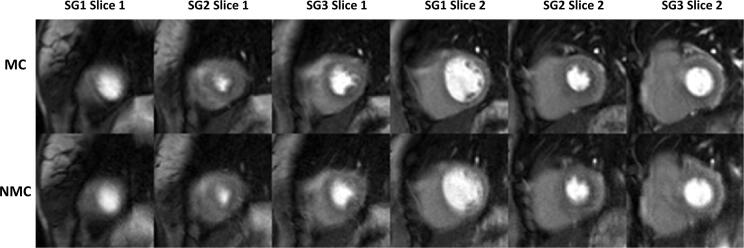
SMS-bSSFP perfusion images at peak myocardial enhancement acquired in a patient with a suboptimal breath-hold (6 uncorrupted first-pass frames) and no perfusion defect. MC images scored higher than NMC in terms of image quality (3.7 vs. 1.7, respectively), motion/blurring (2.7 vs 1.0, respectively) and diagnostic confidence (2.7 vs 1.0, respectively). See Supplementary data 2 for a video of the complete perfusion series.

**Fig. 3:**
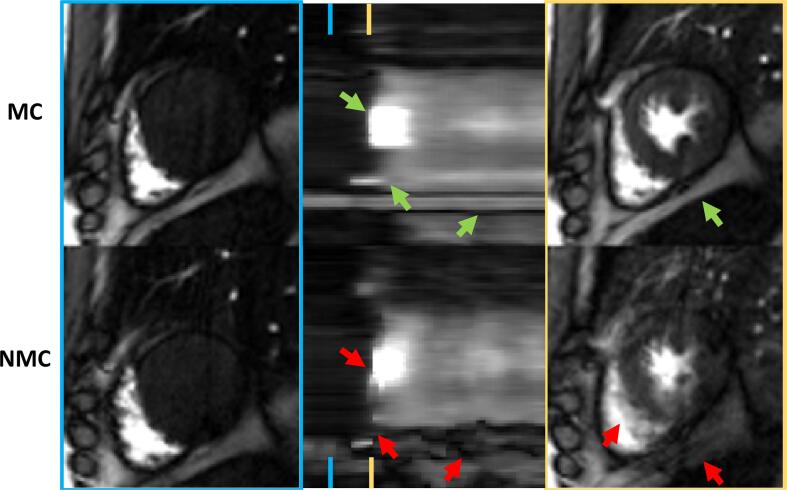
Detailed analysis of the same suboptimal BH case as shown in Fig. 1. The x-t graphs in the centre show that the very short BH (3 dynamic frames) ends directly before the first pass (red arrows on the bottom centre plot). Images on the left show comparable image quality for NMC and MC during the breath hold at baseline. Images on the right show an overlay of multiple respiratory states in the NMC data during the first-pass, while the MC approach was able to align all respiratory states.

**Fig. 4:**
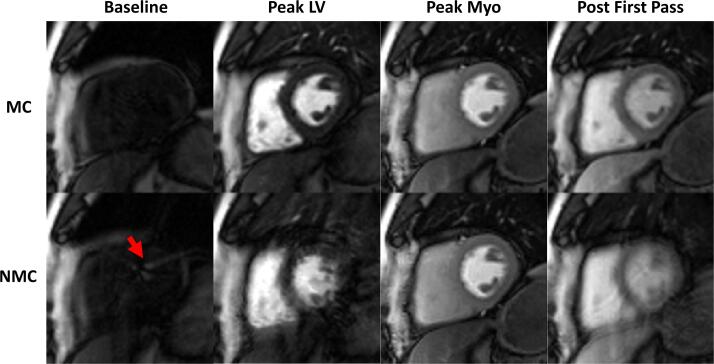
One myocardial slice from a good BH case, shown at four timepoints: baseline, just before the first pass, peak myocardial enhancement and after first pass. In spite of the good BH during the first pass, ghosting and leakage artefacts are visible in the NMC baseline images (red arrow), and images are blurred leading up to and after the peak myocardial enhancement frame. No significant blurring or artefacts are observed for the MC images. See Supplementary data 3 for a video of the complete perfusion series.

Of 31 patients included in the study, 14 patients (45%) with a suboptimal BH (<20 uncorrupted first-pass frames) were identified (0–4 frames: 3, 5–9 frames: 5, 10–14 frames: 1, 15–19 frames: 5). Fig. 1, Fig. 2 show representative SMS-bSSFP images acquired in two patients with a suboptimal BH (3 and 6 uncorrupted first-pass frames, respectively) and reconstructed with and without MC. Videos of these series can be viewed in Supplementary data 1–2. Significant artefacts are observed across all slices of the NMC dataset shown in Fig. 1/Supplementary data 1, while these artefacts are largely absent from the MC reconstruction. Substantial blurring and degraded image quality is observed for both cases reconstructed with NMC, while MC resulted in substantial visual improvement across the entire perfusion series (including frames before/after the first pass; as shown in Supplementary data 1–2). Fig. 3 shows a detailed analysis of the same suboptimal BH case shown in Fig. 1. A line profile through the heart plotted against time demonstrates improved alignment of the left ventricle across the entire perfusion series using the MC reconstruction, which results in reduced temporal blurring of the images acquired across different respiratory phases. Representative images from a good BH case (>20 uncorrupted first-pass frames) are shown in Fig. 4. While image quality is similar for NMC and MC during the first pass BH frames, artefacts and image blurring are present on the NMC dataset outside of this BH segment. The full perfusion series from this case is shown in Supplementary data 3.

**Fig. 5:**
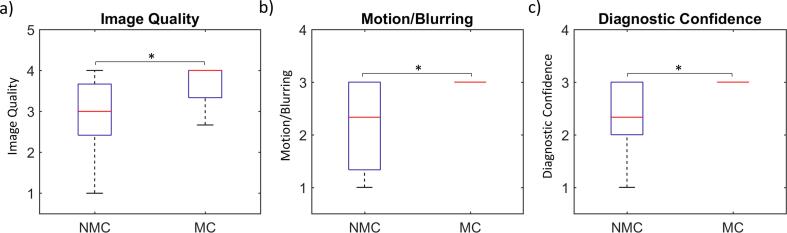
Boxplots showing distribution of a) image quality, b) motion/blurring and c) diagnostic confidence scores for all patients, averaged across the 3 expert readers. On each plot, the red bar represents the median value, the black whiskers represent the minimum and maximum values and the blue box represents the interquartile range (=0 where no box is present). Significant differences are indicated by an asterisk (P < 0.01).

**Fig. 6:**
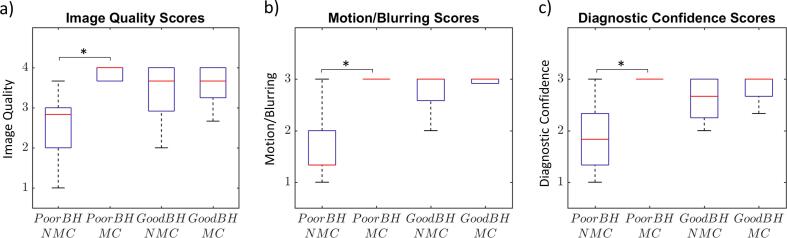
Boxplots showing distribution of a) image quality, b) motion/blurring and c) diagnostic confidence scores for each subgroup of patients, averaged across the 3 expert readers. For a description of the boxplot features, see caption of Fig. 5.

**Fig. 7:**
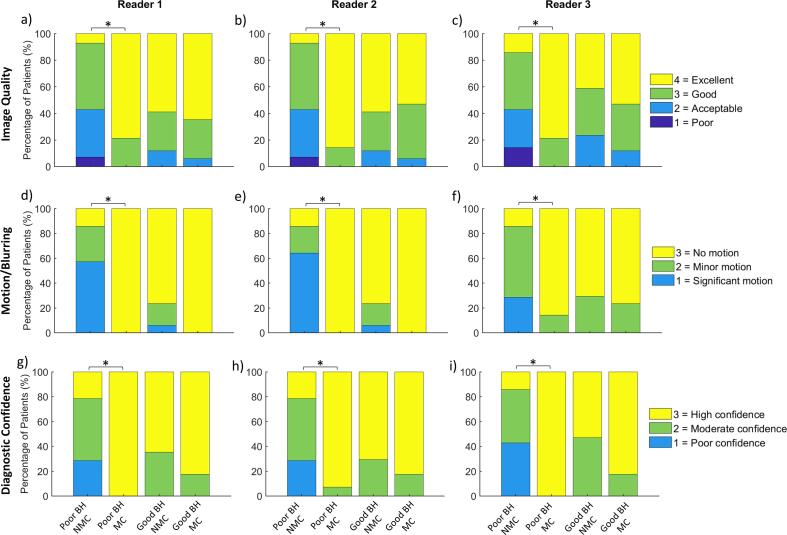
Results of qualitative assessment for each group and each reader. Distribution of scores are displayed as a percentage of the total number of patients in each group. a-c) Image quality scores, d-f) motion/blurring scores and g-i) diagnostic confidence scores. Significant differences are indicated by an asterisk (P < 0.0125).

Results of the qualitative scoring are presented in Fig. 5, Fig. 6, Fig. 7, showing the global comparison, the subgroup comparison (suboptimal BH/good BH), and the subgroup comparison for each reader individually, respectively. Compared across all cases (Fig. 5), MC performed better than NMC in terms of image quality (3.5 ± 0.5 vs. 3.0 ± 0.8, *P =* 0.002), motion/blurring (2.9 ± 0.1 vs. 2.2 ± 0.8, *P <* 0.001), diagnostic confidence (2.9 ± 0.1 vs. 2.3 ± 0.7, *P <* 0.001) and sharpness index (0.34 ± 0.05 vs. 0.31 ± 0.06, *P <* 0.001). The good BH group with NMC reconstruction scored better than the suboptimal BH group with NMC reconstruction in terms of image quality (3.4 ± 0.7 vs. 2.6 ± 0.8, *P =* 0.006), motion/blurring (2.7 ± 0.5 vs. 1.6 ± 0.7, *P <* 0.001) and diagnostic confidence (2.6 ± 0.4 vs. 1.9 ± 0.7, *P =* 0.001). There was no difference in sharpness index (0.31 ± 0.07 vs. 0.30 ± 0.06, *P =* 0.52). For the suboptimal BH group, MC performed significantly better than NMC in terms of image quality (3.8 ± 0.4 vs. 2.6 ± 0.8, *P <* 0.001), motion/blurring (3.0 ± 0.1 vs. 1.6 ± 0.7, *P <* 0.001), diagnostic confidence (3.0 ± 0.1 vs. 1.9 ± 0.7, *P* < 0.001) and sharpness index (0.34 ± 0.05 vs. 0.30 ± 0.06, *P =* 0.004). Comparison between good BH MC and good BH NMC datasets showed no statistically significant differences in image quality (3.5 ± 0.6 vs. 3.4 ± 0.7, *P* = 0.64), motion/blurring (2.9 ± 0.1 vs. 2.7 ± 0.5, *P* = 0.11) or diagnostic confidence (2.8 ± 0.3 vs. 2.6 ± 0.4, *P* = 0.13). However, the sharpness index was higher for the MC dataset than the NMC dataset (0.34 ± 0.06 vs 0.31 ± 0.07, *P* = 0.03). There were no significant differences observed between the suboptimal BH MC and good BH MC datasets in terms of image quality (3.8 ± 0.4 vs. 3.5 ± 0.6, *P* = 0.09), motion/blurring (3.0 ± 0.1 vs. 2.9 ± 0.1, *P* = 0.54), diagnostic confidence (3.0 ± 0.1 vs. 2.8 ± 0.3, *P* = 0.06) or sharpness index (0.34 ± 0.1 vs. 0.34 ± 0.06, *P* = 0.54). There was excellent inter-reader agreement for image quality scores (ICC = 0.91), motion/blurring scores (ICC = 0.94) and diagnostic confidence scores (ICC = 0.90).

## 4 Discussion

This study has demonstrated that integration of motion compensation into a regularised, iterative reconstruction framework for SMS-bSSFP perfusion imaging with high spatial coverage results in improved image quality in the presence of motion and does not degrade motion-free images. The described reconstruction approach significantly increased the image quality, reduced motion/blurring and improved diagnostic confidence of myocardial perfusion images acquired with a suboptimal BH.

While previous studies have demonstrated the benefits of SMS-bSSFP combined with iterative reconstruction compared to conventional perfusion sequences, sensitivity to respiratory motion was recognised as a significant limitation of the technique [10], [11], [12], [26]. Given that 45% of patients with suspected CAD in this study were unable to sustain a BH for the full duration of the first-pass, the results of this study are important for the translation of SMS-bSSFP perfusion imaging into clinical practice. It is further noted that the patients included in this study were coached for optimal breath holding before image acquisition, so the fraction of patients maintaining a good BH in clinical practice may be even lower than observed in this study. This iterative reconstruction with MC framework could also potentially benefit other acquisition techniques using temporal regularisation in the reconstruction process.

The primary motivation for integrating motion compensation into the iterative reconstruction framework was to improve the diagnostic confidence of the perfusion assessment in cases with a suboptimal BH. The data confirms that this has been achieved, and in addition, the motion-compensated reconstruction framework did not reduce the diagnostic confidence in good BH cases. In fact, unlike for the NMC data, motion/blurring scores for the good BH cases with MC virtually all fell into the highest category. One might expect such improvements for cases with good BH due to the overall increased temporal sparsity across the entire acquisition (which is acquired in free-breathing before/after the first-pass), when motion estimation is integrated into the reconstruction. Indeed, the sharpness index was improved for good BH cases reconstructed with MC. While the other three metrics were also higher for the MC reconstruction of good BH cases, these improvements did not reach statistical significance, which may be related to the subtlety of the effect, combined with a relatively small sample size (*n* = 17) and a relatively coarse 3–4 point grading system.

A number of motion-compensated CS reconstruction techniques have been developed for CMR perfusion imaging including schemes which correct for rigid motion [27], [28] or non-rigid motion [13], [17], as well as patch-based algorithms which implicitly correct for motion over neighbouring temporal frames [14], [29]. In the current study, a motion-compensated CS reconstruction, which has been previously demonstrated for single-band gradient-echo perfusion acquisitions [15], was adapted for reconstruction of SMS-bSSFP perfusion imaging. In the context of SMS, the observed improvements using the MC approach are likely to be not just due to MC of the myocardium, but of the whole content of the FOV. Bright signals/signal edges such as fat on the chest or the diaphragm are especially prone to leak between simultaneously acquired SMS slices in the presence of motion and high acceleration using the described SMS framework. Depending on the phase of these ghosts/leaks, they can lead to additional bright signal or signal voids, where the latter could potentially be mistaken for a perfusion defect. Alternative CS reconstruction techniques for SMS perfusion with or without MC have employed outer-volume suppression [30], [31], optimised coil selection [30] and/or regularization which does not rely on temporal redundancy [31] to help to reduce such aliasing artefacts. This is especially critical in motion-compensated techniques which use rigid registration of an ROI around the heart at the expense of motion degradation outside the heart [28], [30].

The motion estimation in this study was performed in a completely separate step, based on images that were reconstructed without any temporal regularisation to ensure a maximum fidelity of the motion states before motion estimation. While this aspect was not evaluated separately, it is likely that such approaches lead to better motion compensation than using temporally regularised images as the basis for motion estimation, e.g. when incorporating the motion estimation and compensation only in the last iteration of the temporally regularised reconstruction as described in [15].

The current study employed a breath-hold approach during the first pass, which is the current clinical standard for CMR perfusion imaging [20]. When the breath-hold is performed well, this approach freezes both in-plane and through-plane motion, the latter of which cannot be corrected for retrospectively. The framework evaluated in this study would be expected to also perform well under free-breathing conditions, however, this needs to be evaluated in future studies including prospective free-breathing exams.

As previously stated, the proposed motion compensation strategy cannot correct for through-plane motion, which can be achieved using prospective slice-tracking with a diaphragmatic navigator [32], [33]. The integration of such a technique in the proposed framework will be the focus of future work.

Another important aspect receiving significant recent attention is the clinical assessment of absolute quantification of myocardial perfusion [34], [35]. Already for traditional quantification approaches [18], [36], [37], a higher number of dynamic frames may be required than for visual assessment, as evaluated in this study, including frames typically acquired in free-breathing (i.e. at baseline before injection of contrast and after the first-pass of contrast agent). Therefore, MC has the potential to improve quantification, in both good and suboptimal BH cases, by compensating for motion in these free-breathing sections of the sequence.

More advanced approaches [19], [38] aim at the quantification of additional parameters such as extracellular volume. These require a much higher number of motion corrected frames which cannot be achieved in the context of a breath hold. Motion-compensated reconstruction techniques may be a way to enable these more complex quantification approaches in the context of highly accelerated, temporally regularised CMR perfusion imaging.

### 4.1 Limitations

This study was carried out in a relatively small cohort of patients, which may have limited the statistical significance of the comparison of qualitative metrics between MC and NMC reconstructions in patients with a good BH. SMS-bSSFP with motion compensation was not compared against a conventional 3-slice perfusion sequence. Therefore, despite significant improvements across all image quality metrics, future studies will be needed to determine if these improvements lead to improved outcome in terms of diagnosis and/or treatment strategy.

## 5 Conclusions

This study compared the performance of an iterative reconstruction framework with and without integrated motion compensation for highly accelerated SMS-bSSFP first-pass perfusion imaging with high myocardial coverage and spatial resolution. The reconstruction incorporating motion compensation demonstrated improved diagnostic confidence and image quality as well as reduced blurring in the presence of respiratory motion.

### CRediT authorship contribution statement

**Sarah McElroy:** Conceptualization, Data curation, Formal analysis, Investigation, Methodology, Project administration, Visualization, Writing – original draft, Writing – review & editing. **Karl P. Kunze:** Conceptualization, Data curation, Investigation, Methodology, Resources, Software, Visualization, Writing – original draft, Writing – review & editing. **Muhummad Sohaib Nazir:** Conceptualization, Data curation, Formal analysis, Funding acquisition, Methodology, Project administration, Resources, Writing – review & editing. **Peter Speier:** Software, Writing – review & editing. **Daniel Stäb:** Software, Writing – review & editing. **Adriana D.M. Villa:** Formal analysis, Writing – review & editing. **Momina Yazdani:** Resources, Writing – review & editing. **Vittoria Vergani:** Resources, Writing – review & editing. **Sébastien Roujol:** Conceptualization, Funding acquisition, Methodology, Supervision, Writing – review & editing. **Radhouene Neji:** Conceptualization, Methodology, Software, Writing – review & editing. **Amedeo Chiribiri:** Conceptualization, Formal analysis, Funding acquisition, Methodology, Resources, Supervision, Validation, Writing – review & editing.

## Declaration of Competing Interest

The authors declare the following financial interests/personal relationships which may be considered as potential competing interests: KPK, RN, PS and DS are employees of Siemens Healthcare. The remaining authors have nothing to disclose.

## Appendix A Supplementary material

Supplementary Data 1 Video showing perfusion images acquired in a patient with a suboptimal BH and reconstructed with MC (top) and without MC (bottom). Significant artefacts are observed across all slices using the NMC reconstruction, while these artefacts are largely absent from the MC reconstruction.

Supplementary Data 2 Video showing perfusion images acquired in a patient with a suboptimal BH and reconstructed with MC (top) and without MC (bottom). Substantial blurring and degraded image quality is observed using the NMC reconstruction, while MC resulted in substantial visual improvement across the entire perfusion series.

Supplementary Data 3 Video showing perfusion images acquired in a patient with a good breath-hold (>20 uncorrupted first-pass frames). Image quality is comparable for NMC and MC during the first pass breath-hold frames. However, there are significant artefacts and image blurring on the NMC dataset outside of this breath-hold segment, whereas the MC images are largely free of artefacts/blurring.
